# Supplementary material for: Cloud BioLinux: pre-configured and on-demand bioinformatics computing for the genomics community
Source: BMC Bioinformatics. 2012 Mar 19;13:42. doi: 10.1186/1471-2105-13-42 (PMC3372431; doi:10.1186/1471-2105-13-42)
Supplement: Additional file 1 — Supplementary 1 Cloud BioLinux software documentation in the form of a mini, self-contained website. Users need to download and uncompress the .zip file, and open through a web browser the "index.html" file available on the main directory. (ZIP 1823 kb). [file 1471-2105-13-42-S1.ZIP › Cloud-BioLinux-Package-Documentation/docs/r.html]

Bio-Linux Software Documentation Pages

  
  
  


Back to search form

### r

|  |  |
| --- | --- |
| Name | r |
| Description | R is a language and environment for statistical computing and graphics. It is a GNU project which is similar to the S language and environment which was developed at Bell Laboratories (formerly AT&T, now Lucent Technologies) by John Chambers and colleagues. R can be considered as a different implementation of S. There are some important differences, but much code written for S runs unaltered under R. R provides a wide variety of statistical (linear and nonlinear modelling, classical statistical tests, time-series analysis, classification, clustering, ...) and graphical techniques, and is highly extensible. The S language is often the vehicle of choice for research in statistical methodology, and R provides an Open Source route to participation in that activity. One of R's strengths is the ease with which well-designed publication-quality plots can be produced, including mathematical symbols and formulae where needed. Great care has been taken over the defaults for the minor design choices in graphics, but the user retains full control. R is available as Free Software under the terms of the Free Software Foundation's GNU General Public License in source code form. It compiles and runs on a wide variety of UNIX platforms and similar systems (including FreeBSD and Linux), Windows and MacOS. |
| Homepage | http://www.r-project.org/ |
| Remote Documentation | http://cran.r-project.org/manuals.html |
